# Identification of Inhibitory Premotor Interneurons Activated at a Late Phase in a Motor Cycle during *Drosophila* Larval Locomotion

**DOI:** 10.1371/journal.pone.0136660

**Published:** 2015-09-03

**Authors:** Yuki Itakura, Hiroshi Kohsaka, Tomoko Ohyama, Marta Zlatic, Stefan R. Pulver, Akinao Nose

**Affiliations:** 1 Department of Complexity Science and Engineering Graduate School of Frontier Sciences, The University of Tokyo, Kashiwanoha, Kashiwa, Chiba, Japan; 2 Janelia Farm Research Campus, Howard Hughes Medical Institute, Ashburn, Virginia, United States of America; 3 Department of Physics, Graduate School of Science, The University of Tokyo, Hongo, Bunkyo-ku, Tokyo, Japan; Center for Genomic Regulation, SPAIN

## Abstract

Rhythmic motor patterns underlying many types of locomotion are thought to be produced by central pattern generators (CPGs). Our knowledge of how CPG networks generate motor patterns in complex nervous systems remains incomplete, despite decades of work in a variety of model organisms. Substrate borne locomotion in *Drosophila* larvae is driven by waves of muscular contraction that propagate through multiple body segments. We use the motor circuitry underlying crawling in larval *Drosophila* as a model to try to understand how segmentally coordinated rhythmic motor patterns are generated. Whereas muscles, motoneurons and sensory neurons have been well investigated in this system, far less is known about the identities and function of interneurons. Our recent study identified a class of glutamatergic premotor interneurons, PMSIs (*period*-positive median segmental interneurons), that regulate the speed of locomotion. Here, we report on the identification of a distinct class of glutamatergic premotor interneurons called Glutamatergic Ventro-Lateral Interneurons (GVLIs). We used calcium imaging to search for interneurons that show rhythmic activity and identified GVLIs as interneurons showing wave-like activity during peristalsis. Paired GVLIs were present in each abdominal segment A1-A7 and locally extended an axon towards a dorsal neuropile region, where they formed GRASP-positive putative synaptic contacts with motoneurons. The interneurons expressed vesicular glutamate transporter (vGluT) and thus likely secrete glutamate, a neurotransmitter known to inhibit motoneurons. These anatomical results suggest that GVLIs are premotor interneurons that locally inhibit motoneurons in the same segment. Consistent with this, optogenetic activation of GVLIs with the red-shifted channelrhodopsin, CsChrimson ceased ongoing peristalsis in crawling larvae. Simultaneous calcium imaging of the activity of GVLIs and motoneurons showed that GVLIs’ wave-like activity lagged behind that of motoneurons by several segments. Thus, GVLIs are activated when the front of a forward motor wave reaches the second or third anterior segment. We propose that GVLIs are part of the feedback inhibition system that terminates motor activity once the front of the motor wave proceeds to anterior segments.

## Introduction

Rhythmic movements, such as walking, swimming, and flying, are commonly driven by neural networks known as central pattern generators (CPGs). CPGs produce rhythmic motor patterns in the absence of sensory feedback [1–3], and are found in many species including insects and mammals, sharing many similarities [4, 5]. CPG networks underlying locomotion exhibit features common to many neural circuits, such as spatio-temporal coordination and flexibility. Each cycle of motor output involves sequences of muscle contraction and relaxation in multiple parts of the body, and can last for a long period (over hundreds of milliseconds to seconds) compared to the time scale of an action potential or synaptic transmission [6–8]. Moreover, the duration of each cycle can change according to the circumstances [9]. In general, CPG networks consist of interconnected interneurons that generate motor patterns underlying rhythmic behaviors. Since interneurons and their neurites are densely packed in the central nervous system (CNS), it has been extremely difficult in many animals to identify these interneurons and clarify their properties and function. However, recent technological innovations have provided powerful tools to tackle this problem, such as novel genetically-coded probes designed to monitor [10–13] or control [14–16] neural activity, and genetic systems for expression of such probes in specific subsets of neurons [17, 18]. Especially in *Drosophila*, more than 1000 Gal4 lines are now available that allow for transgene expression in small subsets of neurons [19]; these resources enable *Drosophila* researchers to characterize the function of single identified interneurons within CPG networks consisting of thousands of neurons.

This study focused on neural networks underlying *Drosophila* larval locomotion and aimed to identify and characterize interneurons that may be involved in regulation of locomotor activity. Forward peristaltic locomotion is the most dominant behavior in *Drosophila* 3^rd^-instar wandering larvae [20]. This stereotyped movement is characterized by waves of muscle contraction that propagate from posterior to anterior segments [21–26]. The CNS of *Drosophila* consists of the brain and the ventral nerve cord (VNC). When the brain is excised or when brain activity is inhibited with genetically-encoded molecular tools, larvae still exhibit peristaltic waves of muscle contraction [21, 25]. Furthermore, while the sensory feedback from muscle contractions controls the speed of the locomotion, sensory inputs are not required to produce the motor patterns [27, 28]. These data suggest that interneurons in the VNC generate the motor pattern, as in other systems [4, 6]. The VNC consists of three thoracic and eight abdominal neuromeres (T1 to T3 and A1 to A8). Motoneurons in each neuromere innervate body-wall muscles in the corresponding or the next posterior body segment. Thus, a forward contraction wave results from the motoneuronal wave-like activity that propagates anteriorly within the VNC. Recordings from the nerve bundles that contain axons of multiple motoneurons revealed three features of the locomotor output [21]. First, the motoneurons exhibit bursting activities. Second, activities of right and left nerves are in phase and those of distinct segments occur sequentially. Third, bursting activities of neighboring segments overlap in time. In addition, a detailed study of crawling in 1^st^-instar intact larvae showed that within a segment, there is a time difference between contraction of ventral/dorsal muscles and that of lateral muscles [26]. These studies suggest that this larval crawling requires spatio-temporal control both within a segment and across multiple segments. Based on these observations, a CPG network model for this locomotion has been offered, where Wilson-Cowan Excitatory-Inhibitory units locating in each neuromere are coupled [29]. However, it is currently difficult to verify such a model, for there is little experimentally-obtained knowledge on actual component neurons and connections. Given these background, it is now of great importance to identify component interneurons in this network and to clarify their activity patterns, connectivity and function.

Our previous study identified a class of glutamatergic premotor interneurons called PMSIs (*period*-positive median segmental interneurons) and revealed their function related to peristaltic locomotion [30]. In this study, we report on the identification and characterization of another class of glutamatergic premotor interneurons, Glutamatergic Ventro-Lateral Interneurons (GVLIs). GVLIs were activated after motoneurons in each neuromere A7-A1, at a specific phase in each of the locomotor cycles. As a whole, GVLIs exhibited a wave-like activity that propagated along segments, following several neuromeres behind the motoneuronal wave. Anatomical analyses suggested that GVLIs release Glutamate (Glu) onto motoneurons in the same neuromere. Since Glu has inhibitory effects on motoneurons in this system [31], GVLIs are, like PMSIs, putative inhibitory premotor interneurons. Consistent with this, optogenetic activation of GVLIs impaired peristaltic locomotion. Taken together, our results suggest that GVLIs suppress motoneuronal activity on the trailing edge of motoneuron bursts during locomotor waves in the larval CNS.

## Materials and Methods

### Flies

Fly stocks were maintained at room temperature (around 25°C). The GAL4/UAS and LexA/lexAop based systems were used for transgene expression [32, 33]. *R26F05-* and *R26A08-Gal4* were generated in the Rubin Laboratory [19]. *RRa-Gal4-F*, one of the *even-skipped Gal4* lines described previously [34], was used to induce transgene expression in aCC (and weak, stochastic expression in RP2) at 3^rd^ instar larval stage. *OK6-Gal4* was described in [35] and *OK6-LexA* was generated from *OK6-Gal4* by replacing the enhancer-trap P-element transgene with *Gal4* with that of *lex* [30]. *OK6-Gal4* drives expression in most if not all motoneurons but only in a few interneurons [36]. *OK6-LexA* largely recapitulates the expression pattern, although the level of expression is lower. We also confirmed by looking at the neuromuscular junctions (NMJs) that most if not all motor axons are labeled by *OK6-LexA* (data not shown). *per-Gal4* was used to induce transgene expression in PMSIs [30]. The following UAS and lexAop lines were used: *10XUAS-IVS-mCD8*::*GFP* [37], *13XlexAop-mCD8*::*GFP*, *10XUAS-IVS-mCD8*::*RFP* [37], *UAS-GCaMP6f* [38], *UAS-syt*::*GFP* [39], *UAS-CD4*::*splitGFP1-10* [40], *lexAop-CD4*::*splitGFP11* [40], *heat-shock Flippase* (*pBPhsFlp2*) [41], *10XUAS>stop>myr*::*GFP* (*pJFRC41*) [37], *20XUAS-CsChrimson-mVenus* [16], and *mhc-GFP* [28]. *Canton-S* was used as a wild-type control.

### Calcium Imaging

Larvae expressing GCaMP6f [38] were dissected in an external solution containing: (in mM) 135 NaCl, 5 KCl, 4 MgCl_2_, 2 CaCl_2_, 5 TES (N-tris [hydroxymethyl] methyl-2-aminoethane sulfonic acid), 36 sucrose and adjusted to pH 7.1 with NaOH [42]. The CNS was isolated by cutting nerve bundles and surrounding tissues and anchored to a sylgard-coated dish by pinning down residual tissue. Such immobilized preparations enable us to observe neural activities at high magnification, while lack of sensory feedback slows down the propagating speed of motor activity. We used an Axioskop2 FS microscope (Zeiss, Germany) with a 40X water immersion objective lens and EMCCD camera (iXon, Andor, Germany) to acquire image data. A spinning-disk confocal unit (CSU21, Yokogawa, Japan) was used to monitor GVLIs’ and PMSIs’ activity simultaneously to minimize the overlap of the neurites in a vertical (dorsal-ventral) direction. 720 images were acquired with exposure time for each image set to 250 milliseconds without extra interval, thus neural activities for ~3 minutes were recorded for each preparation. To present the images as pseudocolor images, we applied a look-up table “Royal” to image stacks using ImageJ software (NIH). For time-course analysis, the mean fluorescent intensity in each region of interest (ROI) was normalized and represented as ΔF/F. Peak times of intensity increase were read out from the graphs and analyzed. For regression analysis, the coefficient of determination, r^2^ and the p-value by test for significance of the regression were calculated by using Microsoft Excel.

### Immunohistochemistry and imaging

Dissected larvae were fixed and stained according to a previous study [43] with a few modifications. Briefly, the samples were fixed in 4% formaldehyde diluted with phosphate buffered saline (PBS) at 4°C for 30 min, washed repeatedly with PBS, placed in PBS with 0.2% TritonX-100 (PBT), in PBT with normal goat serum, and incubated with primary antibodies overnight at 4°C. After rinses, secondary antibodies were applied. Primary antibodies used in this study are as follows: rabbit anti-GFP (Frontier science, Af2020, 1:1000), mouse anti-Fasciclin2 (Fas2) (DSHB, 1D4, 1:10), rabbit anti-vGluT (vGluT: vesicular glutamate transporter) [44] (1:1000), mouse anti-ChAT (ChAT: Choline acetyltransferase) (Hybridoma Bank, 4B1, 1:50), rabbit anti-GABA (Sigma-Aldrich, A2052, 1:100), rabbit anti-DsRed (Clontech, no. 632496, 1:500), guinea pig anti-GFP (Frontier science, Af1180, 1:1000), Cy5-conjugated goat anti-Horseradish Peroxidase (HRP) (Life Technologies, 1:200). Secondary antibodies used are Alexa488-conjugated goat anti-rabbit IgG (Life Technologies, 1:300), Cy5-conjugated goat anti-mouse IgG (Life Technologies, 1:300), Cy3-conjugated goat anti-rabbit IgG (Life Technologies, 1:300), Alexa555-conjugated goat anti-mouse IgG (Life Technologies, 1:300), Alexa488-conjugated goat anti-guinea pig IgG (Life Technologies, 1:300). The preparation was fixed for 10 min when GFP signal was detected without immunolabeling. For imaging and analysis, confocal laser scanning microscope and its software (Fluoview 1000, Olympus, Japan) with water-immersion 20x, 60x and 100x objective lenses were used. Imaris software (Bitplane) was used to reconstruct the collected horizontal images and acquire cross-sectional images.

### Genetic mosaic method

Adult virgin females with genotype of *10XUAS>stop>myr*::*GFP* and adult males *hsFLP; R26F05-Gal4* were crossed and put in a container covered with agar plate with yeast paste. The offspring eggs on the plate were collected for two-hour intervals and allowed to develop for 3 hours before heated to 36°C for an hour in an incubator. The embryos were put back to 25°C, and 4 days later when the animals developed into late 3^rd^ instar wandering larvae, we dissected them and performed immunostaining to label the expressed GFP, endogenous Fas2 and vGluT.

### Locomotion analyses

For CsChrimson experiments, flies were crossed and kept in vials with food containing 1 mM all-trans retinal (ATR) with minimum exposure to light, and the resulting offspring were used. Experiments were performed at room temperature (25±1°C) in a dimly lit room to minimize activation of CsChrimson by ambient light. In each trial, a 3^rd^ instar wandering larva was put in deionized water to remove extraneous matter. The larva was then placed on an agar plate with a diameter of 9 cm. Larval movements were recorded with a CCD camera (XCD-V60, Sony) under a microscope (MVX10, Olympus, Japan). Images were obtained at a rate of 15–30 frames per second and saved as audio video interleaving (avi) files. To image behavior, larvae were exposed to dim white light (~2 μW / mm^2^) throughout the recording. During a muscle contraction wave (in the posterior two thirds of the body), larvae were exposed to red light (660 nm LED, Thorlabs, USA) with an intensity of around 30~40 μW/mm^2^ for several seconds. For quantitative analysis, the percentage of larvae in which the muscle contraction wave was disrupted upon light illumination was measured. To statistically analyze the data, we used Fisher’s exact probability test with Bonferroni correction. dTRPA1 experiments and analyses were performed according to a previous report [45]. Peltier module (Oven Industries PA, Model 0805) for controlling temperature was controlled through the MWT software (http://sourceforge.net/projects/mwt). An aluminium plate (10 x 10 cm) covered with a thin layer of 3% charcoal agar was placed on the Peltier module. The temperature of the agar was raised from 20°C to 32°C, to activate the dTRPA1 channel. The time to reach the target temperature ranged from 3 to 8 s. Larvae containing the *UAS-dTRPA1* transgene were raised at 18°C and 3^rd^ instar larvae were used for the experiments. 15–25 larvae were placed on the agar gel at once. The temperature of the entire rig was kept at room temperature (22°C– 25°C). The speed of crawling stride is calculated by average of all speed of stride in crawling events using LARA software [45]. To calculate the instantaneous percentage of crawling larvae in responses to thermogenetic activation, the animals that were tracked throughout the entire -10 sec to 75 sec (0 sec = start temperature sift to 32°C from 20°C) were selected for analysis. Then, mean of a speed of crawling stride or percentage of crawling animals are calculated at each time point from crawling events. Percent of crawling time and the speed of crawling stride were obtained from the data between 65 sec and 75 sec. Statistical analysis was performed using MATLAB (MathWorks) software. Percent of crawling time and the speed of peristaltic crawling strides were compared using Wilcoxon rank sum test and Bonferroni correction for multiple comparisons.

### Local optogenetic activation

Larvae were dissected to expose the CNS and body-wall in an external solution used for calcium imaging. We applied local blue light illumination (488nm) by Ar laser and monitored muscle contraction waves under confocal microscopy (Fluoview 1000, Olympus, Japan) with a CCD camera (XCD-V60, Sony) and 4x objective lens. Dissection and imaging was performed under dim white light (~2 μW / mm^2^).

### Wave phase analyses

We introduced *wave duration*, which represents the time difference between the peak of aCCs’ activity in A6 (t_aCC_(A6)) and that in T3 neuromere (t_aCC_(T3)), as a measure of a duration of a motor wave:

$$wave\;duration={\text{ t}}_{\text{aCC}}\left(\text{T3}\right){\;-\text{ t}}_{\text{aCC}}\left(\text{A6}\right)$$

Only the activities in A6-T3 segments were considered, since those in more posterior and anterior segments could not be reproducibly recorded. The *wave duration* varied from 4 to 18 seconds and the average ± s.e.m was 9 ± 1 seconds (n = 19). We defined the *wave phase (P)* by setting t_aCC_(A6) and t_aCC_(T3) as 0% and 100%, respectively. The peak times of aCCs’ and GVLIs’ activities in each neuromere were converted to percentage of the *wave phase*:

$$\begin{array}{l} P_{\text{aCC}}\left(\text{An}\right)\;=\;\left({\text{t}}_{\text{aCC}}\left(\text{An}\right){\;-\text{ t}}_{\text{aCC}}\left(\text{A6}\right)\right)\;/\;\left({\text{t}}_{\text{aCC}}\left(\text{T3}\right){\;-\text{ t}}_{\text{aCC}}\left(\text{A6}\right)\right)\;\left(\%\right)\\ P_{\text{GVLI}}\left(\text{An}\right)\;=\;\left({\text{t}}_{\text{GVLI}}\left(\text{An}\right){\;-\text{ t}}_{\text{aCC}}\left(\text{A6}\right)\right)\;/\;\left({\text{t}}_{\text{aCC}}\left(\text{T3}\right){\;-\text{ t}}_{\text{aCC}}\left(\text{A6}\right)\right)\;\left(\%\right) \end{array}$$

## Results

### Identification of GVLIs by calcium imaging

**Fig 1:**
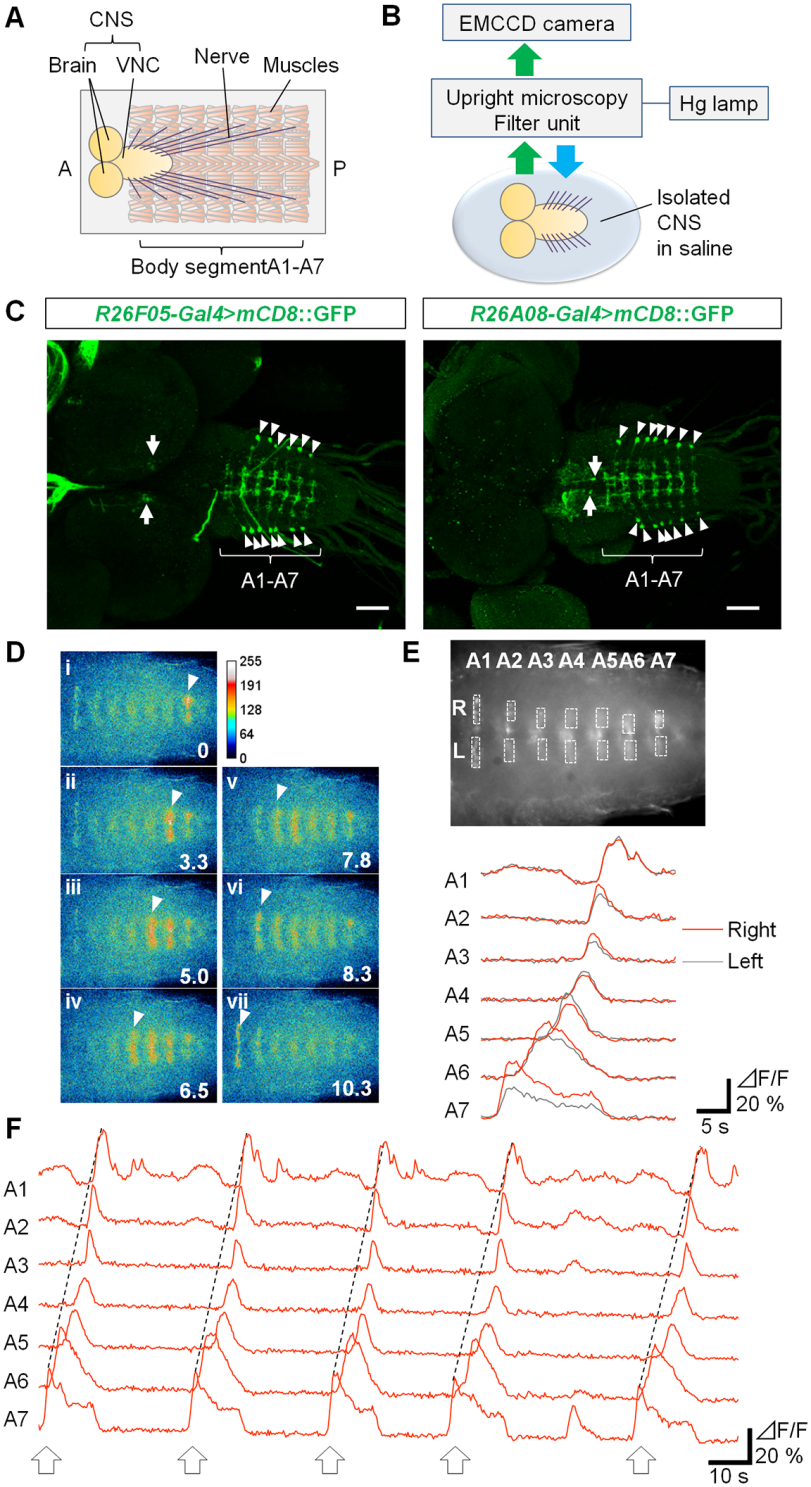
Identification of GVLIs and their wave-like activity. (A) Anatomical chart of the 3^rd^ instar larva. (B) Experimental set-up for calcium imaging. (C) Expression of GVLI Gal4 drivers, *R26F05-Gal4* (left) or *R26A08-Gal4* (right), visualized with mCD8::GFP. Expression in a pair of GVLIs in each of abdominal neuromeres A1 to A7 is shown by arrowheads. *R26F05-* and *R26A08-Gal4* also induces expression in neurons in the brain and the SOG and thoracic neuromeres (arrows), respectively. Scale bars represent 50μm. (D) Neural activity of GVLIs monitored by calcium imaging in the VNC of a *R26F05-Gal4*>*UAS-GCaMP6f* larva. Images obtained at the peak activity of neurites in each of the neuromeres A7 (time: 0) to A1 (time: 10.3 seconds) were shown in i to vii, respectively. GCaMP signal was pseudocolored. Arrowheads indicate the neurites exhibiting peak activity. (E) The regions of interest were set as shown in the upper panel (neurites locating in the right and left hemineuromeres in A1 to A7) and the intensity change in each ROI was plotted in the lower panel, showing a wave-like activity with simultaneous activation in right (orange lines) and left (gray lines) side of the VNC. (F) Representative data from a sample. ROIs were set at the right side. Arrows indicate forward waves. The third wave is same as waves shown in (D) and bottom panel of (E).

Although the *Drosophila* 3^rd^ instar larval VNC consists of a relatively small number of neurons compared to the vertebrate spinal cord, it still is a highly complex structure containing cell bodies and neurites from ~10000 neurons [46, 47]. Therefore, to identify classes of interneurons involved in larval crawling and to clarify how they function in the neural circuit, it is important to target a specific class of neurons reproducibly. To make this possible, we used the GAL4/UAS transgenic system to express various molecular probes in specific candidate interneurons. As interneurons that exhibit wave-like activity similar to motor activity generating muscle contraction waves are likely to play important functional roles in this locomotor network, we used calcium imaging to search for Gal4 lines which drive expression in interneurons that show such activity patterns. We expressed the genetically encoded calcium indicator GCaMP6f [38] and imaged fluorescence signal of GCaMP6f in isolated CNS preparations (Fig 1A and 1B), and identified several classes of interneurons which showed wave-like activity. In this study, we focus on one of them, termed GVLIs, a pair of neurons in each abdominal segment in A1-A7, which were identified by two independent Gal4 lines, *R26F05-* and *R26A08-Gal4*. Both of the two Gal4 lines drive expression only in GVLIs in the abdominal VNC, although they also drive expression in small numbers of cells in the brain (in the case of *R26F05-Gal4*) and subesophageal ganglion (SOG) and in thoracic segments (in the case of *R26A08-Gal4*) (Fig 1C). Fig 1D–1F shows the activity pattern of GVLIs revealed by calcium imaging experiments. GVLIs in each neuromere were activated sequentially from A7 to A1 (Fig 1D–1F). The strongest fluorescence increase was seen in the neurites of GVLIs located in a dorsomedial region of the VNC. The neurites locating on the left and right side in a neuromere were activated synchronously (Fig 1E). We therefore analyzed only the right side of the VNC in the following experiments. The fact that the wave-like activity of GVLIs was observed in the isolated CNS suggests that the activity pattern can be generated by the central circuits in the absence of sensory feedback.

### GVLIs form glutamatergic terminals in a dorsomedial region in the neuropile

**Fig 2:**
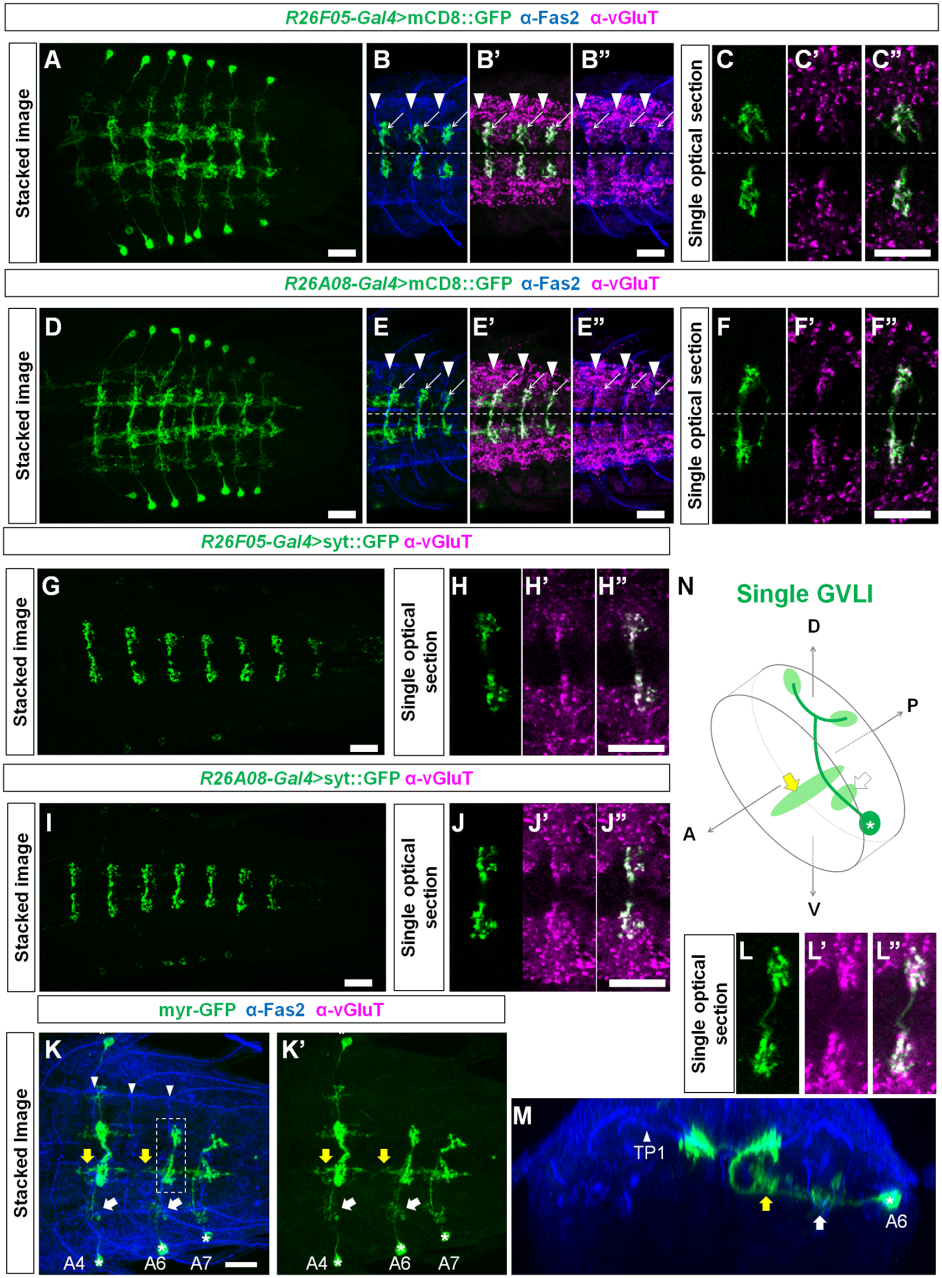
Morphology and anatomical properties of GVLIs. (A-F) Morphology of GVLIs as visualized with mCD8::GFP expressed under the control of *R26F05-Gal4* (A-C) or *R26A08-Gal4* (D-F). (A, D) Stacked images of the whole VNC, dorsal view. (B, E) Enlarged views of dorsal region of the neuropile in A3 to A5, co-stained for Fas2 (blue) and vGluT (magenta). Arrowheads indicate TP1 projections. vGluT localization was observed in the neurites of GVLIs (arrows). (C, F) Localization of VGluT in neurites of GVLIs in a single optical section of 1.11 μm thickness. (G-J) Localization of Syt::GFP in GVLIs. Expression driven by *R26F05-Gal4* (G, H) or *R26A08-Gal4* (I, J). (G, I) Stacked images of the VNC. Syt::GFP staining is largely confined to the dorso-medial neurites of GVLIs. (H, J) Colocalization of Syt::GFP with vGluT. (K-N) Mosaic labeling of GVLIs with myr-GFP (K-M) and a diagram of a single GVLI (N). (L) Putative axon terminals are seen at both ipsi- and contralateral side (enclosed by dotted lines in K). Yellow arrows and white arrows indicate putative dendritic sites. One of them in a medial position (yellow arrows) extended to the anterior neuromere (yellow arrows, beyond TP1 projections (arrowheads)). The cell body is indicated by asterisk. Scale bars, 20 μm.

To characterize neurotransmitter phenotype of GVLIs, we used three antibodies, anti-vGluT (vesicular glutamate transporter), anti-ChAT (choline acetyltransferase) and anti-GABA (γ-amino butyric acid), markers for glutamatergic, cholinergic and GABAergic neurons, respectively. We observed co-localization of vGluT signals with neurites of GVLIs (as visualized with mCD8::GFP, Fig 2A and 2D) (Fig 2B', 2C, 2E' and 2F) but not those of ChAT or GABA (data not shown), suggesting that GVLIs are glutamatergic. Expression of vGluT was seen in GVLIs’ neurites in the dorsomedial region of the neuropile (Fig 2B and 2E). We also examined the expression of a presynaptic marker, GFP-tagged synaptotagmin (syt::GFP) in GVLIs and detected expression of syt::GFP at the vGluT-positive site (Fig 2G–2J). Since vGluT is known to accumulate at presynaptic sites of glutamatergic neurons and loads glutamate into synaptic vesicles, it is likely that GVLIs release Glu in the dorsomedial region. This site also exhibited the strongest calcium signals during the wave-like activity, consistent with it being a presynaptic site. In summary, these results suggest that GVLIs release Glu from terminals in the dorsomedial region in the neuropile.

We next used mosaic analysis to study the morphology and projection patterns of single GVLIs (Fig 2K–2M, see Materials and methods for details on the production of single clones). From the cell body (located lateral to the VL tract; tract nomenclature according to [48]), a neurite extended medially and then dorsally and finally innervated the dorsomedial region where the vGluT-positive putative axon terminals were observed. Terminals are located in the anterior commissures near the intersection with longitudinal connectives. Single GVLIs were found to innervate these sites both ipsi- and contralaterally (Fig 2K, see GVLIs in A6 and A7) via V-shaped axons that project in the anterior commissure (along a tract immediately posterior to TP1 projections, S1 Fig). Terminals on both sides showed VgluT staining (Fig 2L). From the main axon branch, two neurite extensions were seen, one along the CL and the other along a longitudinal axis between CI and DM tract (indicated by white and yellow arrows, respectively in Fig 2K, 2M and 2N and S1 Fig). It is most likely that these two neurite extensions contain postsynaptic sites, as no notable syt::GFP signal was observed in these regions. The neurite extension in the medial region projected anteriorly, crossed the segment boundary and extended into the next anterior neuromere (15 out of 15 GVLI clones, see yellow arrows in Fig 2K), suggesting a possibility that the neurites receive information from anterior neuromere(s).

### GVLIs are glutamatergic premotor interneurons

**Fig 3:**
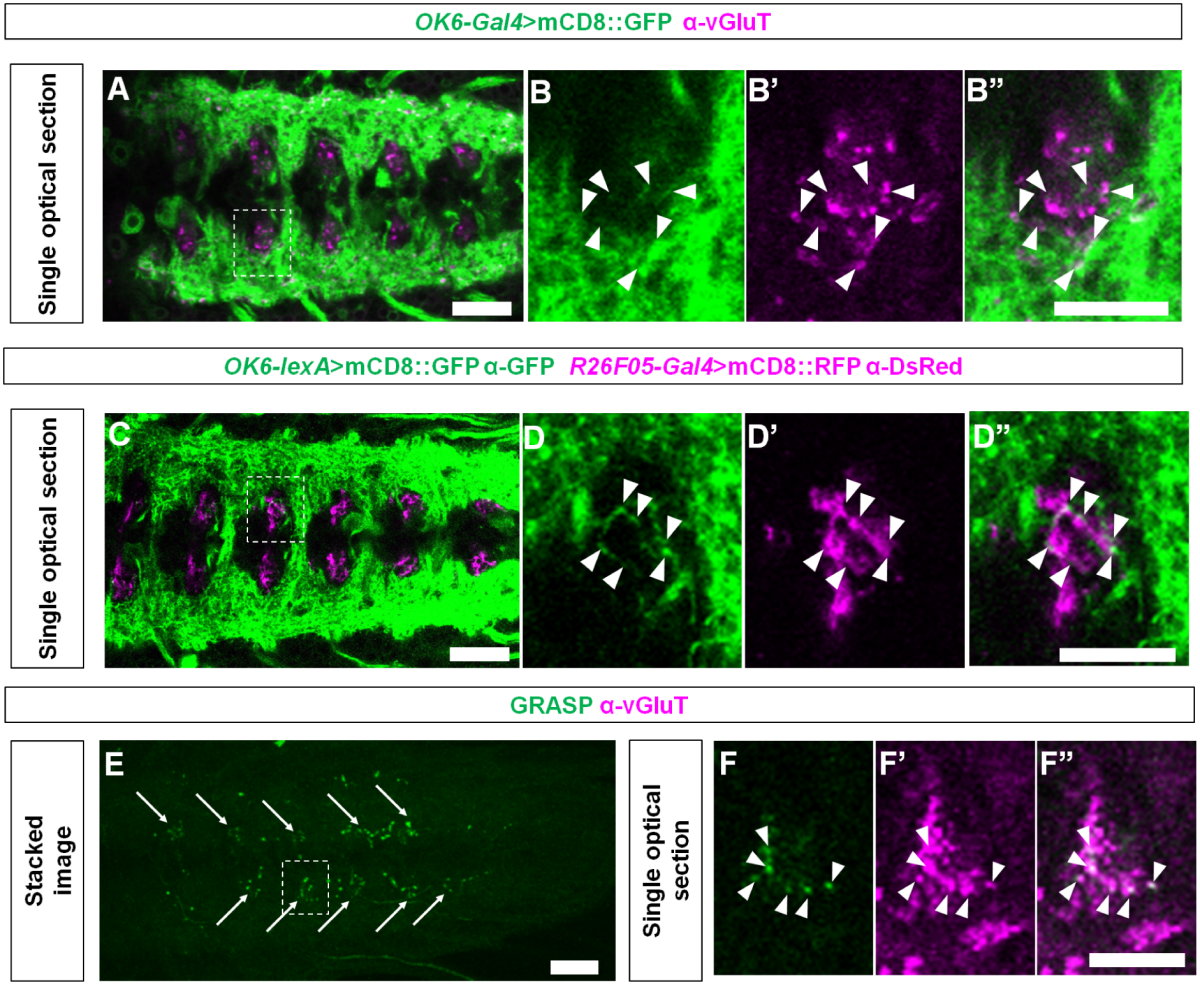
Putative synaptic connection between GVLIs and motoneurons. (A) Dendrites of motoneurons and axon terminals of GVLIs are visualized with mCD8::GFP driven by *OK6-Gal4* and endogenous vGluT expression, respectively. Scale bar, 20 μm. The region enclosed by dotted lines in (A) was enlarged in (B). Arrowheads indicate sites where GFP and vGluT signals are adjacent to each other. Scale bar, 10 μm. (C) Motoneurons and GVLIs are visualized with mCD8::GFP and mCD8::RFP driven by *OK6-lexA* and *R26F05-Gal4*, respectively. Scale bar, 20 μm. The region enclosed by dotted lines in (C) was enlarged in (D). Arrowheads indicate sites where GFP and RFP signals are in proximity. Scale bar, 10 μm. (E) A stacked image of GRASP signals obtained with *OK6-LexA* and *R26F05-Gal4*. The signals are seen segmentally (arrows). Scale bar, 20 μm. (F) Colocalization of the GRASP signals with vGluT (arrowheads) in a single optical section (enlarged view of the region indicated by dotted rectangle in E). Scale bar, 10 μm.

Motoneuron neurites within the VNC are largely restricted to the dorsal area and sensory projections to the ventral area in the neuropile [48]. As GVLIs’ putative axon terminals are located dorsally in the neuropile, GVLIs could be pre-synaptic to motoneurons. To test this possibility, we first studied the proximity of motoneuronal dendrites (visualized with GFP driven by *OK6-Gal4*, a Gal4 line specific to motoneurons [36]) and GVLI axon terminals (visualized with anti-vGluT antibody. Note that terminals of GVLI can be distinguished from other vGluT-positive boutons based on their unique positions and morphology) (Fig 3A). Several motoneuronal dendrites were observed adjacent to the GVLI axon terminals within a single optical section of 0.77 μm thickness obtained by confocal microscopy (Fig 3B). We also labeled motoneurons and GVLIs with different colors using *OK6-LexA* and *R26F05-Gal4* and observed close apposition between the GVLI axon terminals and motoneuronal dendrites (Fig 3C and 3D). As detailed in Materials and methods, *OK6-LexA* largely recapitulates the expression pattern of *OK6-Gal4*, although the level of expression is lower (S2 Fig). To examine further the putative connections between motoneurons and GVLIs, we employed the GFP reconstitution across synaptic partners (GRASP) system [40, 49]. When we expressed split GFP 1–10 and split GFP 11 using *R26F05-Gal4* and two copies of *OK6-LexA*, respectively (Fig 3E), we detected GRASP signals and the vGluT-positive signals (in presumptive axon terminals of GVLIs) colocalized within single optical sections of confocal microscopy (Fig 3F) (6 out of 6 larvae). No signal was detected with one copy of *OK6-LexA* (0 out of 7 larvae) or in the absence of *R26F05-Gal4* (0 out of 6 larvae), indicating the specificity of the GRASP signals (data not shown). These findings suggest that GVLIs could be premotor interneurons which release glutamate as a transmitter. While both PMSIs and GVLIs are glutamatergic putative premotor interneurons, the terminals of GVLIs and PMSIs are located in distinct regions in the neuropile (S3 Fig top panel), suggesting that they innervate different sets of motoneurons and/or distinct dendritic branches of the same motoneurons. Since glutamate is known to have inhibitory effects on motoneurons in this system [31], it is most likely that GVLIs in a given neuromere directly inhibit motoneurons in the same neuromere.

### Optogenetic activation of GVLIs disrupts crawling behavior

**Fig 4:**
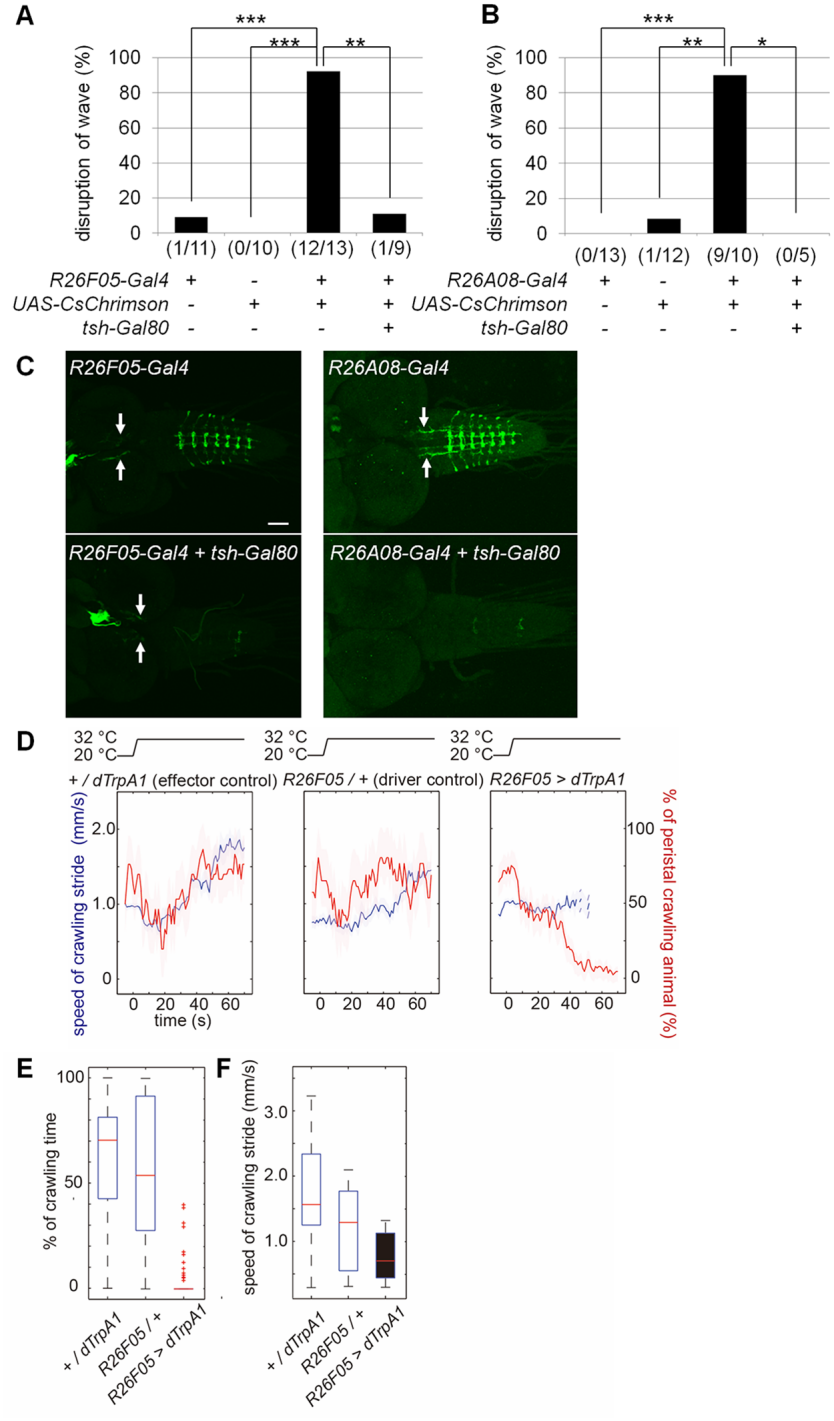
Locomotion deficits caused by activation of GVLIs. (A, B) Disruption of muscle contraction waves by optogenetic activation of *R26F05-Gal4* (A) or *R26A08-Gal4* (B) expressing neurons with CsChrimson and reversion of the phenotype by *tsh-Gal80*. 660 nm red light was applied to larvae exhibiting a muscle contraction wave and the percentage of larvae whose contraction wave ceased was measured for *R26F05-Gal4*>*UAS-CsChrimson* (A) or *R26A08-Gal4>UAS-CsChrimson* (B) and the control groups. n = 11, 10, 13 and 9 larvae in (A) and 13, 12, 10 and 5 larvae in (B). ***p<0.001; **p<0.01; *p<0.05 (Fisher’s exact probability test with Bonferroni correction). (C) CsChrimson expression patterns under the control of each Gal4 line (upper panels) and suppression of the expression in the VNC with *tsh-Gal80* (bottom panels) as visualized with mVenus immunostaining. Arrows indicate expression in other cells than GVLIs. (D-F) Thermal dTRPA1 activation of GVLIs greatly slows or terminates crawling behavior. (D) Graphs show the time course of speed of crawling stride and percentage of animals crawling before and during thermal dTRPA1 activation. The red line shows the percentage of larvae crawling at each time point. The speed of crawling strides is also shown for those animals crawling at each time point (blue line). Dark lines show means; shaded areas indicate standard errors. The top panel indicates the temperature change during the experiment. The temperature starts to rise toward the permissive temperature (34°C) at 0 s and reaches it in 3 to 5 s. Data are from *+/dTRPA1*, *R26F05/+*, and *R26F05>dTRPA1* animals. (E, F) Figures show box plots of (E) the percentage of time animals spent crawling and (F) speed of crawling strides during the 10-s period from 55 to 65 s after the onset of thermal activation. The red line shows the median; the bottom and top edges of each box are the 25^th^ and 75^th^ percentiles, red cross marks show outliers, respectively.

**Fig 5:**
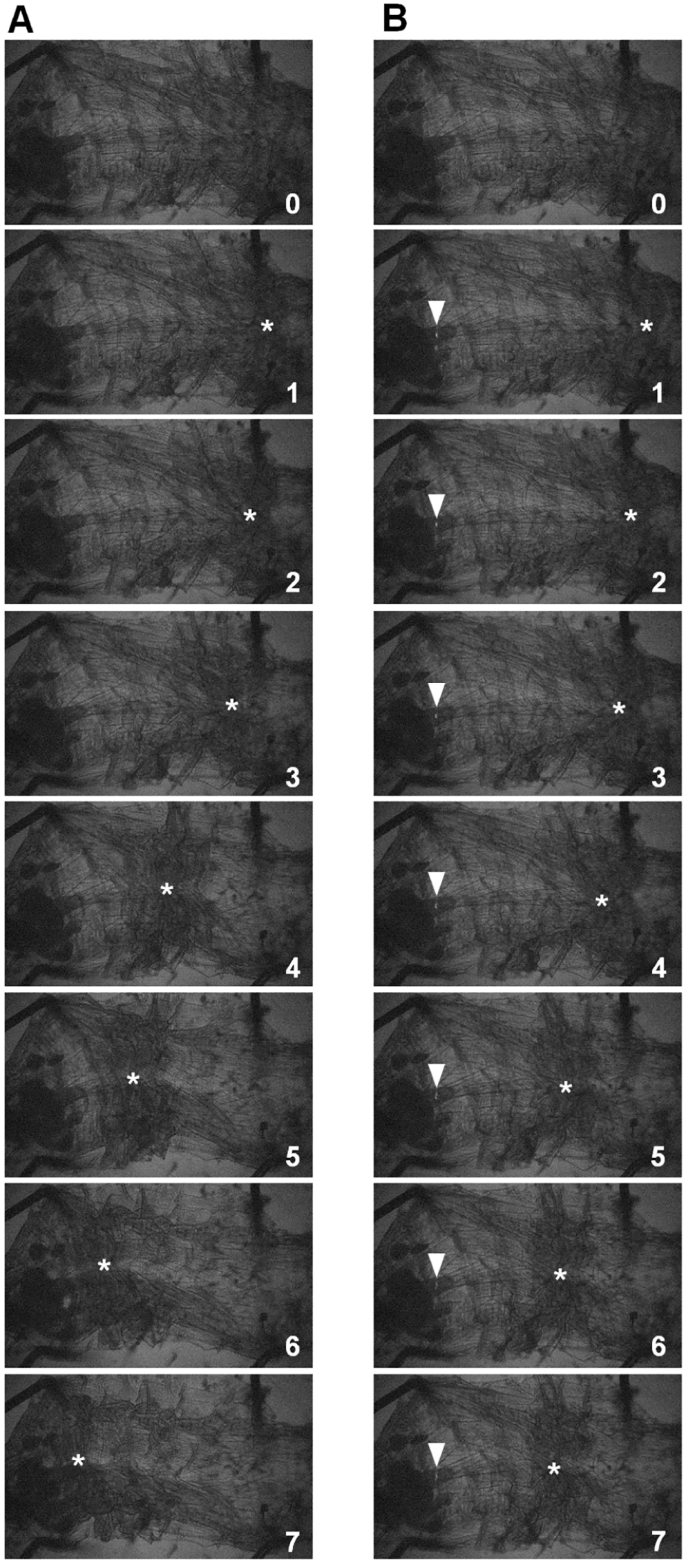
Activation of GVLIs inhibits muscle contraction locally. Muscle contraction waves without (A) and with (B) local illumination with blue (488nm) light (indicated by arrowheads) in the dissected larvae expressing CsChrimson under the control of *R26F05-Gal4*. Numbers displayed at the lower right corner of each image indicate the time (sec). Asterisks indicate the position of muscle contraction in the body wall. Propagation of muscle contraction is arrested upon light application at a position roughly corresponding to the stimulated neuromere(s).

If GVLIs have a functional role in crawling behavior, perturbation of their normal activity may cause deficits in locomotion. To test this, we remotely activiated GVLIs with CsChrimson, a red-shifted version of Channelrhodopsin [16]. Activation of CsChrimson triggers cation influx into neurons, resulting in depolarization and consequent neurotransmitter release at axon terminals. We used CsChrimson instead of channelrhodopsin 2 (ChR2) because of the following two advantages: 1) the wavelength of light used to activate CsChrimson is largely invisible to larvae and does not evoke strong behavioral responses in wild-type animals [50] and 2) light-evoked responses in CsChrimson expressing neurons are stronger than responses in ChR2 expressing neurons [16]. When 660 nm light was applied to behaving larvae expressing CsChrimson in GVLIs, peristaltic waves ceased and the entire body relaxed (S1 Movie, Fig 4A and 4B). Since GVLIs are the only class of neurons marked by both *R26F05-Gal4* and *R26A08-Gal4* and the expression of the two Gal4 lines in other cells than GVLIs is weak and limited to a small number of cells in the brain, SOG and thoracic segments, it is most likely that GVLIs were responsible for the phenotype. To further confirm this, we suppressed GAL4-mediated expression in the VNC by using *tsh-Gal80* [51] (Fig 4C, bottom panels) and observed reversion of the phenotypes for both of the Gal4 lines (Fig 4A and 4B). GVLIs are the only VNC cell type present in the *R26F05-Gal4* expression pattern. Therefore, our results suggest that GVLIs were responsible for the behavioral phenotype and support the idea that GVLIs’ outputs have inhibitory effects on motoneurons. To further confirm this, we used dTRPA1 to activate GVLIs and observed a similar phenotype with *R26F05-Gal4*: a decrease in the proportion of crawling animals and the speed of crawling (Fig 4D–4F). The phenotype was not seen when *R26A08-Gal4* was used as a driver line, possibly because expression level induced by *R26A08-Gal4* is weaker than that of *R26F05-Gal4* (data not shown). Since GVLI axons project to the dendritic domain of motoneurons in the same neuromere, they are likely to inhibit motoneurons locally. We tested this possibility by applying local photo-stimulation to dissected larvae expressing CsChrimson in GVLIs. When the light was applied to a small region spanning a few neuromeres in the VNC of a dissected larva undergoing peristalsis, the peristalsis was arrested in body-wall segments that corresponded to the illuminated neuromeres (Fig 5, S2 and S3 Movies; 8 out of 11 experimental larvae (with *R26F05-Gal4* and *UAS-CsChrimson*) versus 0 out of 10 effector control larvae (with *UAS-CsChrimson*); p = 0.0008, Fisher’s exact probability test). These observations are consistent with the idea that GVLIs function to inhibit motoneurons locally.

Inhibiting activity in GLVIs using Shibire^ts^ [52], Archaerhodopsin [53] and tetanus toxin light chain [54] did not result in defects in basic crawling behavior. This could be due to incomplete silencing of GVLIs, degeneracy within larval locomotor networks or simply reflect the subtle role that GVLIs play in regulating larval locomotion. In addition, there is a possibility that GVLIs contain other co-transmitters (e.g. neuropeptides) in addition to glutamate, whose secretion is not affected by Shibire^ts^, Archaerhodopsin or tetanus toxin light chain. A final possible explanation is that GVLIs are connected to other neurons via electrical synapses and that the functioning of these synapses is not affected by the genetic tools used to inhibit activity in GLVIs.

### GVLIs’ wave-like activity follows motoneuronal wave-like activity in a phase dependent manner

**Fig 6:**
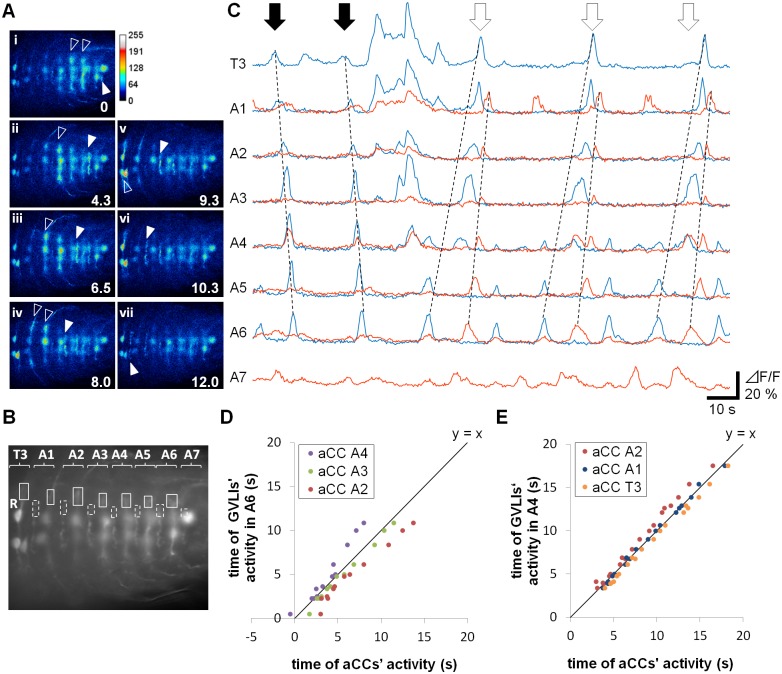
Timing relationship between the wave-like activities of GVLIs and motoneurons. (A) Expression of GCaMP6f with *R26F05-Gal4* (in GVLIs) and *RRa-Gal4-F* (in aCC and RP2 motoneurons). Images obtained during a wave were shown in order of time (i to vii). Values indicate time from (i). GCaMP signal was pseudocolored “Royal.” White and open arrowheads indicate active neurites of GVLIs and aCCs, respectively. (B) Dotted and solid rectangles show ROIs for GVLI and aCC, respectively, used for the calcium imaging analyses shown in (C). (C) Representative data from a sample. Fluorescent intensity change in an aCC (blue) and GVLIs (orange) were plotted. GVLIs were recruited in forward waves (white arrows) but not in backward waves (black arrows). (D, E) The timing of activation of GVLIs and aCCs in the anterior segments were plotted on vertical and horizontal axis, respectively. The time of activation of aCCs in A6 was set as time 0. (D) GVLIs in A6 versus aCCs in A4 to A2; (E) GVLIs in A4 versus aCCs in A2 to T3.

GVLIs are putative inhibitory premotor interneurons that exhibited wave-like activity similar to motoneuronal activity during larval peristalsis. Next we studied the time correlation between the activities of GVLIs and motoneurons to investigate how GVLI activity is related to motor function. For this purpose, we expressed GCaMP6f both in GVLIs and the motoneurons, aCC and RP2, in the same larvae (*UAS-GCaMP6f / +; R26F05-Gal4 / RRa-Gal4*) and observed activities of the two groups of neurons simultaneously. Since the positions of the neurites of GVLIs and aCCs do not overlap, activities of these neurons could be readily distinguished (Fig 6A–6C). While this suggest that aCCs are unlikely to be postsynaptic partners of GVLIs, recording from aCCs provides a good measure for correlating GVLIs activity with motoneuronal activity associated with peristaltic locomotion. In all of the 19 forward motor waves with varying speeds recorded from five larvae, waves of aCC activity were always accompanied by GVLI activation (Fig 6C, white arrows). However, GVLIs were activated later than the motoneurons in the same neuromere and at a similar timing with motoneurons in neuromeres 2–3 segments more anterior (Fig 6A–6E). In other words, activity propagation of GVLIs lagged behind that of motoneurons by 2 or 3 segments. During backward motor waves, GVLIs exhibited no obvious activity pattern (Fig 6C, black arrows), suggesting that GVLIs are not strongly recruited during backward peristaltic activity.

**Fig 7:**
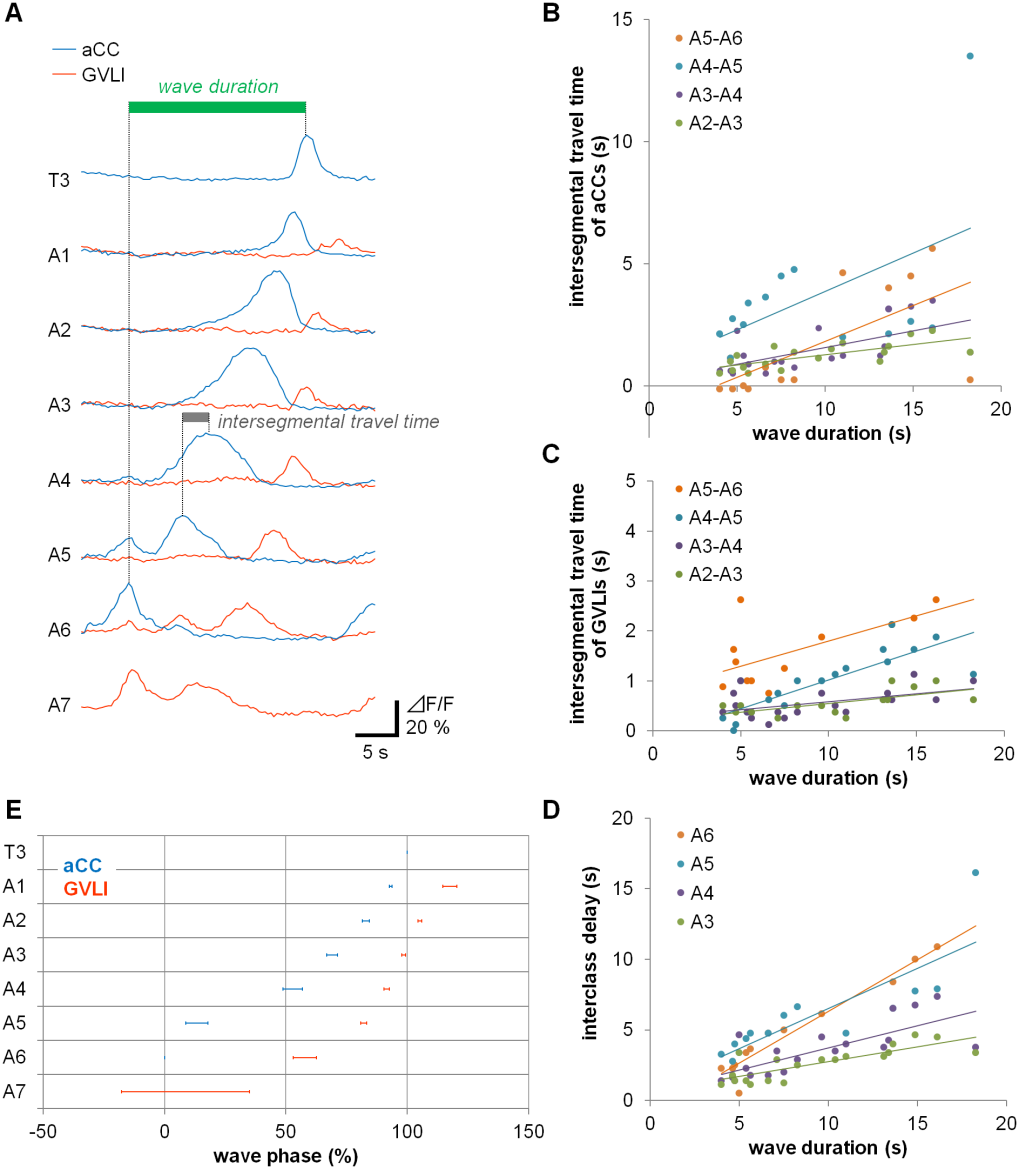
Wave phase analysis of the activity of GVLIs and motoneurons. (A) A representative plot of the activity of aCC (blue) and GVLIs (orange) showing the definition of *wave duration* (green bar) and *intersegmental travel time* (gray bar represents A5-A4 *intersegmental travel time* of aCCs’ activity). (B, C) *Intersegmental travel time* of aCCs (B), GVLIs (C) and delay between activity peaks in aCC and GVLI in a neuromere (D) was plotted as a function of *wave duration* (data obtained from 13–19 waves for (B), 12–19 waves for (C) and (D)). (E) Phase representation of aCCs’ and GVLIs’ activities. The average values ± s.e.m of *P*_aCC_ (An) and *P*_GVLI_ (An) are shown as blue and orange bars, respectively. Interclass phase difference was smaller in anterior neuromeres.

The fact that GVLIs are activated at a similar, but not exactly the same, timing as that of aCC motoneurons in more anterior segments at varying speeds of peristalsis suggest that activity of GVLIs is regulated by intersegmental circuits that coordinate the activity of motoneurons and interneurons along the segments. To further characterize the timing of GVLIs’ activity in relationship to motoneuronal activity during a forward wave, we next investigated relative timing of aCC and GVLI activity across different wave speeds. A previous study [26] showed that intersegmental activation delays in identified muscles scale linearly in 1^st^ instar larvae. We first wanted to confirm if this is also the case for motoneuronal activity in the isolated 3^rd^ instar CNS by studying relative timing of aCC motoneuronal activity in different segments during forward waves. We defined *wave duration* (green bar in Fig 7A) as the time difference between the peak of aCCs’ activity in A6 neuromere (t_aCC_(A6)) and that in T3 neuromere (t_aCC_(T3)) (only the activities in A6-T3 segments were considered since those in more posterior and anterior segments could not be reproducibly recorded). The *intersegmental travel time*, time for activity to propagate from one aCC neuron to the next, was calculated using peak times in neighboring segments (t_aCC_(A(n))–t_aCC_(A(n+1))) (A(n) refers to n^th^ abdominal segment, gray bar in Fig 7A). When *intersegmental travel times* of aCC activity were plotted against *wave duration*, we found linear relationships in all segments. (Fig 7B; the regression linear equation, coefficient of determination and probability value by test for significance of the regression for each were as follows: A2-A3 (y = 0.084x+0.43, r^2^ = 0.50, p < 0.001), A3-A4 (y = 0.14x+0.20, r^2^ = 0.39, p < 0.005), A4-A5 (y = 0.31x+0.73, r^2^ = 0.24, p < 0.1 (not significant)), A5-A6 (y = 0.29x-1.1, r^2^ = 0.43, p < 0.02). Similarly, *intersegmental travel time* of GVLI activity (t_GVLI_(A(n))–t_GVLI_(A(n+1))) increased linearly with *wave duration* in most segments (Fig 7C: A2-A3 (y = 0.035x+0.20, r^2^ = 0.43, p < 0.01), A3-A4 (y = 0.032x+0.26, r^2^ = 0.26, p < 0.05), A4-A5 (y = 0.12x-0.13, r^2^ = 0.70, p < 0.001), A5-A6 (y = 0.10x+0.80, r^2^ = 0.42, p < 0.05). As expected, delay between activity peaks in aCC and GVLI also scaled with *wave duration* in a linear fashion (Fig 7D: A3 (y = 0.21x+0.64, r^2^ = 0.65, p < 0.001), A4 (y = 0.32x+0.58, r^2^ = 0.57, p < 0.001), A5 (y = 0.57x+0.82, r^2^ = 0.68, p < 0.001), A6 (y = 0.73x-0.99, r^2^ = 0.94, p < 0.001)). In 5 out of 6 segments the y intercept of linear regression lines of delay versus *wave duration* were not significantly different from zero, indicating proportional scaling and phase constancy.

Having characterized how aCC and GVLI activity scaled with *wave duration*, we compared average phases of activity in aCC an GVLI: we defined the *wave phase (P)* by setting t_aCC_(A6) and t_aCC_(T3) as 0% and 100%, respectively, and converted the activity of aCC and GVLIs in each neuromere to percentage of the *wave phase* as shown in Fig 7E. In this plot, activity of aCCs and GVLIs were found to be assigned to narrow windows in a motor cycle despite the variance of *wave duration*. The phase representation confirmed the time relationship between the activities of the two neurons described above: in most segments, GVLIs were activated at a similar phase as aCCs in the second or third anterior neuromere. Thus, both GVLIs and aCC motoneurons are activated in a phase dependent-manner with a near-constant phase lag between their activities over different speeds of peristalsis. We also studied the temporal relationship between PMSIs and GVLIs. Consistent with the previous observation [30] showing that PMSIs are activated slightly later than motoneurons with a time delay of ~0.5 neuromeres, we found that activity propagation of GVLIs lagged behind that of PMSIs by ~2 neuromeres during forward waves (S3 Fig; 21 out of 21 waves from 4 *per-Gal4/UAS-GCaMP6f; R26F05-Gal4/+* larvae).

### Muscles in multiple segments contract at a given time during peristaltic waves

**Fig 8:**
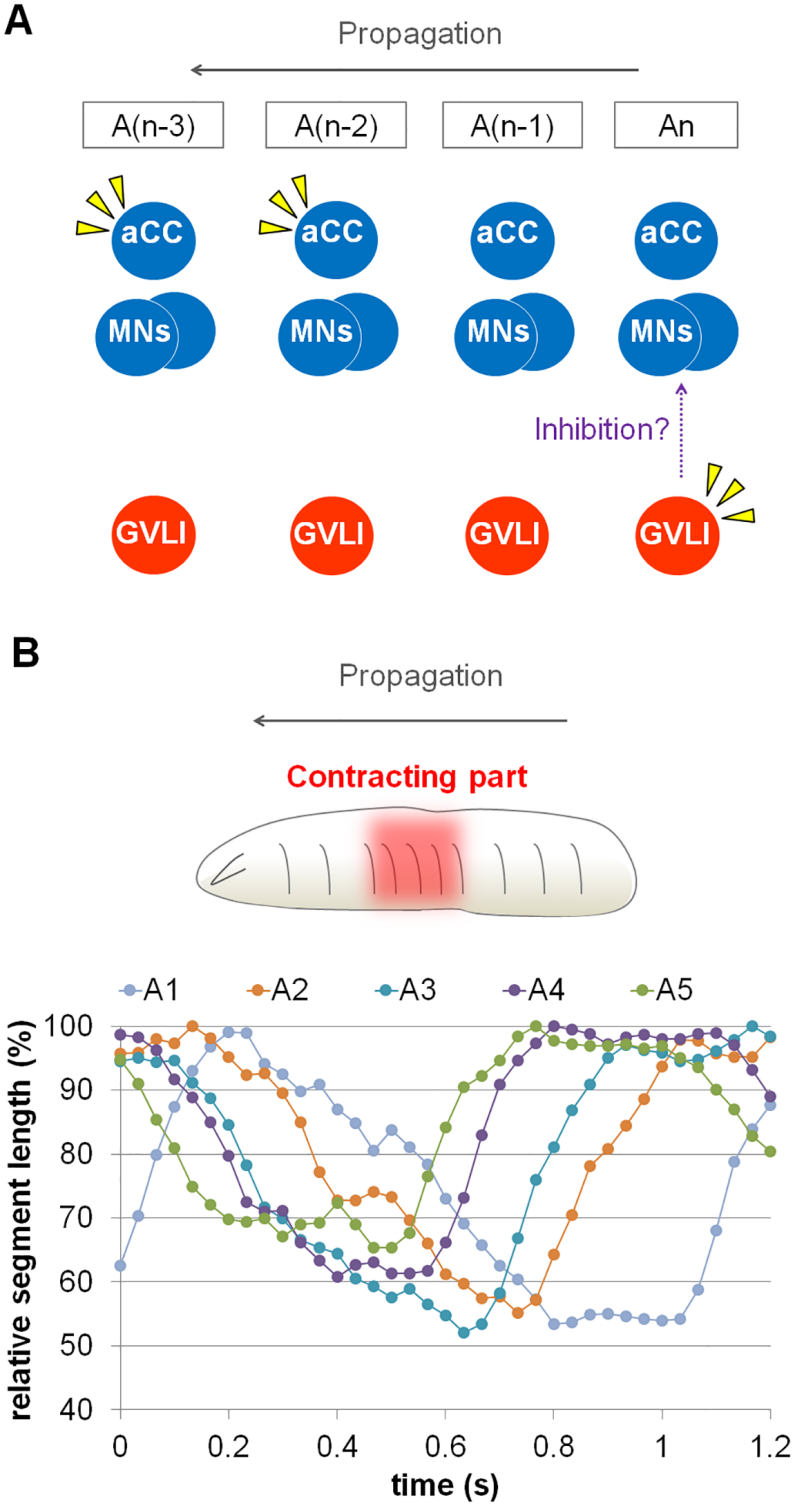
GVLIs’ delayed activation and muscle contraction pattern during peristaltic locomotion. (A) Schematic diagram of the relationship between GVLIs and motoneurons. Yellow arrowheads indicate active neurons. During a forward wave, motoneurons are activated sequentially from posterior to anterior neuromere, in this diagram from An to A(n-3). GVLIs in An neuromere are activated at a similar timing when aCC motoneurons in the second or third anterior neuromere A(n-2) or A(n-3) are active, and inhibit motoneurons in An. (B) (upper panel) Diagram of a muscle contraction wave. Muscles in several consecutive segments contracted simultaneously (red colored), which is quantified and shown in the bottom panel. (bottom panel) Muscle contraction was studied by measuring the longitudinal length of each segment (corresponding to the length of longitudinal muscles) during a step of larval crawling.

The above findings indicate that GVLIs are likely to inhibit motoneurons in the same neuromere when the leading edge of the motor wave has reached the second or third neuromere anterior to a given GVLI (Fig 8A). Thus, one possible function of GVLIs in this motor circuit is to feed back the information of successful propagation of motor wave to more posterior neuromeres. For such feedback inhibition to be meaningful, motoneurons have to be still in an active state when this feedback inhibition occurs. In other words, motoneurons in three or more segments have to be in an active state at a given time. A previous study [26] suggested that it is indeed the case in the 1^st^ instar larvae. We studied whether this is also true in 3^rd^ instars by observing muscle contraction patterns in larvae expressing GFP in muscles (*mhc-GFP* [28]). We examined muscle contraction by measuring the change in segment length during peristaltic propagation and found that muscles in three to six segments are indeed contracting at any given time during peristaltic waves (Fig 8B). Thus, the delayed inhibition by GVLIs is well positioned to terminate motoneuronal activity in the larval locomotor network. However, future experimental testing is required to show that GVLIs indeed function in a manner proposed by the above model.

## Discussion

In this study, we characterized GVLIs, a segmentally repeating interneuron in neuromere A7 –A1 which is active during fictive locomotion in the larval CNS. Anatomical and behavioral analyses suggested that they connect with and inhibit motoneurons in the network. Analyses of activity timing relative to motor activity showed that GVLIs are activated with a distinct timing delay relative to aCC motoneurons. Based on these results, we discuss possible roles of the interneurons.

### GVLIs are putative inhibitory premotor interneurons

The motoneurons involved in *Drosophila* larval peristaltic locomotion are known to be responsive to at least three neurotransmitters, excitatory acetylcholine and inhibitory GABA and glutamate [31]. Therefore, motoneurons likely generate rhythmic motor outputs by integrating multiple inputs. In order to clarify how interneurons contribute to the generation of motoneuronal rhythmic activity, it is essential to identify premotor interneurons and determine how they control the activity of motoneurons. In this study, we identified GVLIs as putative premotor interneurons in this system.

Four lines of evidence suggest that GVLIs are inhibitory premotor interneurons. First, GVLIs express vGluT, a vesicular transporter of glutamate, and thus likely secrete glutamate, a neurotransmitter known to elicit inhibitory responses in motoneurons. Second, vGluT-positive GVLI axon terminals are present in the dorsal region of the neuropile in the vicinity of motoneurons’ dendrites in the same segment [48]. Third, GVLIs form GRASP-positive putative synaptic contacts with motoneurons, although uncertainty remains as to the identity of the target motoneurons. The contact sites express the presynaptic markers Synaptotagmin and vGluT and show robust increases in calcium concentration during peristaltic waves, strongly suggesting that they are presynaptic terminals. Fourth, optogenetic activation of GVLIs inhibited motor function. Activation of GVLIs in crawling larvae disrupted ongoing peristaltic waves. Local activation of GVLIs in dissected larvae halted peristaltic waves in the corresponding region in the body wall. These results are consistent with the idea that GVLIs send inhibitory inputs locally to motoneurons. Taken together, our anatomical and functional analyses strongly suggest that GVLIs are premotor local interneurons that inhibit motoneurons in the same segment. It should be noted, however, that we have not examined if GVLIs form synaptic connections with interneurons. Thus, it remains possible that GVLIs innervate some interneurons in addition to motoneurons. It is also important to note that axon terminals of GVLIs cover only a small portion of the dendritic region of motoneurons and thus likely innervate only a small subset of motoneurons. Considering the strong effect of GVLIs activation, GVLIs may well inhibit a large number of motoneurons via other interneurons.

In *Drosophila*, several glutamate receptors (GluR) have been identified, such as metabotropic GluRs (DmGluR) [55], AMPA/kinate receptor homologues, N-methyl-D-aspartate (NMDA) receptor homologues [56], and glutamate-gated chloride channels (GluCl) [57]. Thus Glu can have various effects on postsynaptic cells depending on the receptors expressed. For instance, Glu causes excitatory junction currents (EJCs) when released at neuromuscular junction (NMJ) [58] and induces hyperpolarizing responses in antennal lobe neurons [59]. Rohrbough and Broadie [31] showed that glutamate application elicits inhibitory responses in larval motoneurons. The effect is blocked by the chloride channel blocker picrotoxin, suggesting the existence of GluCl on motoneurons. Thus it is most likely that GVLIs inhibit motoneurons via GluCl. It should be noted, however, that the inhibitory effects of glutamate via GluCl has only been examined in subsets of motoneurons. It should also be noted that GVLIs may secrete other neurotransmitters in addition to Glu and/or transmit information through gap junctions. Future identification of the postsynaptic partners of GVLIs and the receptors expressed on the cells will provide more information on how GVLIs regulate the activity of downstream motoneurons.

### Phase relationships among the activities of muscles, motoneurons and GVLIs

We used calcium imaging to characterize the activity of GVLIs and aCCs in T3-A7 segments and the activity timing relationships among them. We found that during forward locomotor waves, GVLIs are activated at a similar timing as are aCC neurons in the second or third more anterior neuromeres and later than aCC neurons in the same segment. The phase delay between GVLI and aCC activity remained relatively constant over wide range of *wave durations*. The identity of the postsynaptic motoneuron(s) of GVLIs remains to be determined. However, the axon terminals of GVLIs are located in a neuropile region occupied by dendrites of motoneurons that innervate dorsal/ventral muscles and are activated at the same timing as aCCs. GVLIs therefore are likely to be activated with a delay of 2–3 segments to their target motoneurons. It should be noted, however, the delay would be shorter if the target motoneurons are those innervating lateral muscles since they are known to be activated later than those innervating ventral/dorsal muscles [26].

By studying the activity of aCCs and GVLIs during peristalsis at varying speeds, we showed that phase delays between the two neurons remain relatively constant over a range of *wave durations* as in many undulatory movements spanning multiple body segments [60, 61]. Our results conform to the previous study [26] that showed phase constancy based on the observation of muscle movements. The phase representation of the activation of aCCs and GVLIs shown in Fig 7E, composite data derived from multiple larvae undergoing peristalsis at different speeds, well recapitulated the sequential activation from posterior to anterior segments observed in a single larva. Thus, use of the phase representation is adequate in the analyses of neural activity in this system. The phase delay data indicates that GVLIs, like motoneurons, are regulated by intersegmental networks that maintain phase constancy over different speeds of peristalsis. Although GVLIs were activated at a similar time as aCCs in the second or third anterior neuromere, they were not active at exactly the same time as aCC neurons. This suggests that upstream partners of GVLIs are different from those of motoneurons.

### Possible functions of GVLIs in the larval locomotor network

The onset and termination of muscle contraction must be finely regulated to generate efficient forward movement during larval locomotion [26]. Excitatory and inhibitory premotor neurons active at distinct phases of larval locomotion are likely to be involved in this regulation. During forward locomotion, muscles in three or more segments are simultaneously contracted at a given time ([26] and this study). This indicates that muscle activity is shut down when the front of a muscle contraction wave reaches the third or more anterior segment. The activity pattern of GVLIs revealed by calcium imaging (phasic activation with a two-to-three segment delay compared to aCC motoneurons) is consistent with a role for GVLIs in this process. The anatomy of GVLIs is also consistent with a role in feedback inhibition: each GVLIs extend their putative dendritic processes to anterior neuromeres and their axonal processes to motoneurons in the same segment (Fig 2K–2N). GVLIs may thus inhibit motoneurons and help to terminate muscle contraction when the motor wave reaches the anterior segments, by integrating information from anterior segments and transmitting the signal to motoneurons in the same segment. Whether GVLIs indeed play essential roles in this process remains to be determined since our functional analyses with currently available neural silencers failed to show any obvious phenotypes. It should also be noted that if GVLIs do play such a role, they should only be part of the system since their axonal terminals do not cover the entire dendritic field of motoneurons and thus likely innervate only a subset of motoneurons.

In an independent study, we have recently identified another class of premotor inhibitory neurons PMSIs (*period*-positive median segmental interneurons) [30]. Like GVLIs, PMSIs are glutamatergic and inhibit motor function when activated, and show wave-like activity during peristalsis. However, they are activated at a different phase from that of GVLIs ([30] and this study). They are activated much earlier than GVLIs, shortly after the activation of the postsynaptic motoneurons with a time delay of ~0.5 neuromere, and control the duration of motor bursting and the speed of locomotion. Thus, PMSIs appear to provide early-cycle inhibition that is critical for determining the duration of motor bursting. In contrast, GVLIs may contribute to late-cycle inhibition that terminates motor bursting. Future studies will elucidate how GVLI, PMSI and other premotor interneurons, active at distinct phases of a motor cycle, shape the motor pattern. For example, optogenetic activation of the interneurons can be combined with patch-clamp recordings in motoneurons to study how the activity manipulation changes the pattern of motor activity. Such analyses will pave the way for understanding how rhythm is generated during larval locomotion.

## Supporting Information

S1 Fig Positional relationship among GVLIs’ neurites, the anterior commissure and TP1 projection. (upper two panels) The morphology of GVLIs (visualized with mCD8::GFP expressed under the control of *R26F05-Gal4*, green) in relation to the anterior commissure (white arrows) and posterior commissure (open arrows) labeled with anti-HRP antibody (magenta). A2-A6 neuromeres of the ventral nerve cord are shown. Asterisks indicate the position of A4 anterior commissure. Scale bar, 20 μm. (bottom two panels) The morphology of GVLIs (green, labeled as in upper panels) in relation to the Fasciclin2-positive axon tracts (blue, tracts indicated by white arrowheads). The yellow and white arrows correspond to those in Fig 2K, 2M and 2N.
(TIF)

S2 Fig Expression driven by *OK6-lexA*. (left panels) mCD8::GFP and mCD8::RFP were expressed by one copy of *OK6-lexA* and *OK6-Gal4*, respectively. All of the *OK6-lexA*-expressing (mCD8::GFP-positive) neurons also expressed *OK6-Gal4* (mCD8::RFP-positive) (white and yellow arrowheads; yellow arrowheads indicate the sites where mCD8::RFP was observed in a single optical section, but not in the stacked images shown here). (right panel) mCD8::GFP expression induced by two copies of *OK6-lexA*. The expression pattern is similar to that of *OK6-Gal4*, with no evident expression in interneurons. Scale bar, 20μm.
(TIF)

S3 Fig Temporal relationship between the activity of GVLIs and PMSIs. The time courses of GCaMP6f signal intensity change in the neurites of GVLIs (the dotted rectangles and orange lines) and PMSIs (the solid rectangles and green lines) in neuromeres A3-A7 are shown. Activity propagation of GVLIs lagged behind that of PMSIs by ~2 neuromeres during forward waves (white arrows). PMSIs but not GVLIs were activated in a wave-like manner during backward waves (black arrows). ROIs used for the recording are shown in the upper panel. In this representative example, ROIs for GVLIs were set in the right side of the VNC and those for PMSIs were in the left side to minimize the overlap.
(TIF)

S1 Movie Disruption of muscle contraction waves by activation of *R26F05-Gal4* neurons with CsChrimson. A control (*CsChrimson/+*) or *R26F05>CsChrimson* larva was illuminated with 660 nm red light in the middle of peristaltic locomotion. “ON” or “OFF” in the movie shows the presence or absence of light application, respectively. In the larva expressing CsChrimson in *R26F05-Gal4* neurons, muscle contraction waves were ceased upon light application. The larva resumed crawling after a second or so despite ongoing light illumination, likely due to adaptation of CsChrimson in response to red light [16].
(MP4)

S2 Movie Local activation of *R26F05-Gal4* neurons with CsChrimson prevented the propagation of muscle contraction wave (example 1). *R26F05>CsChrimson* larva was dissected and the muscle contraction waves were recorded. When a few neuromeres in the VNC were illuminated, the muscle contraction wave was arrested at the corresponding segment.
(MP4)

S3 Movie Local activation of *R26F05-Gal4* neurons with CsChrimson prevented the propagation of muscle contraction wave (example 2). (MP4)
