# Supplementary figures and images for: Identification of Inhibitory Premotor Interneurons Activated at a Late Phase in a Motor Cycle during Drosophila Larval Locomotion
Source: PLoS One. 2015 Sep 3;10(9):e0136660. doi: 10.1371/journal.pone.0136660 (PMC4559423; doi:10.1371/journal.pone.0136660)

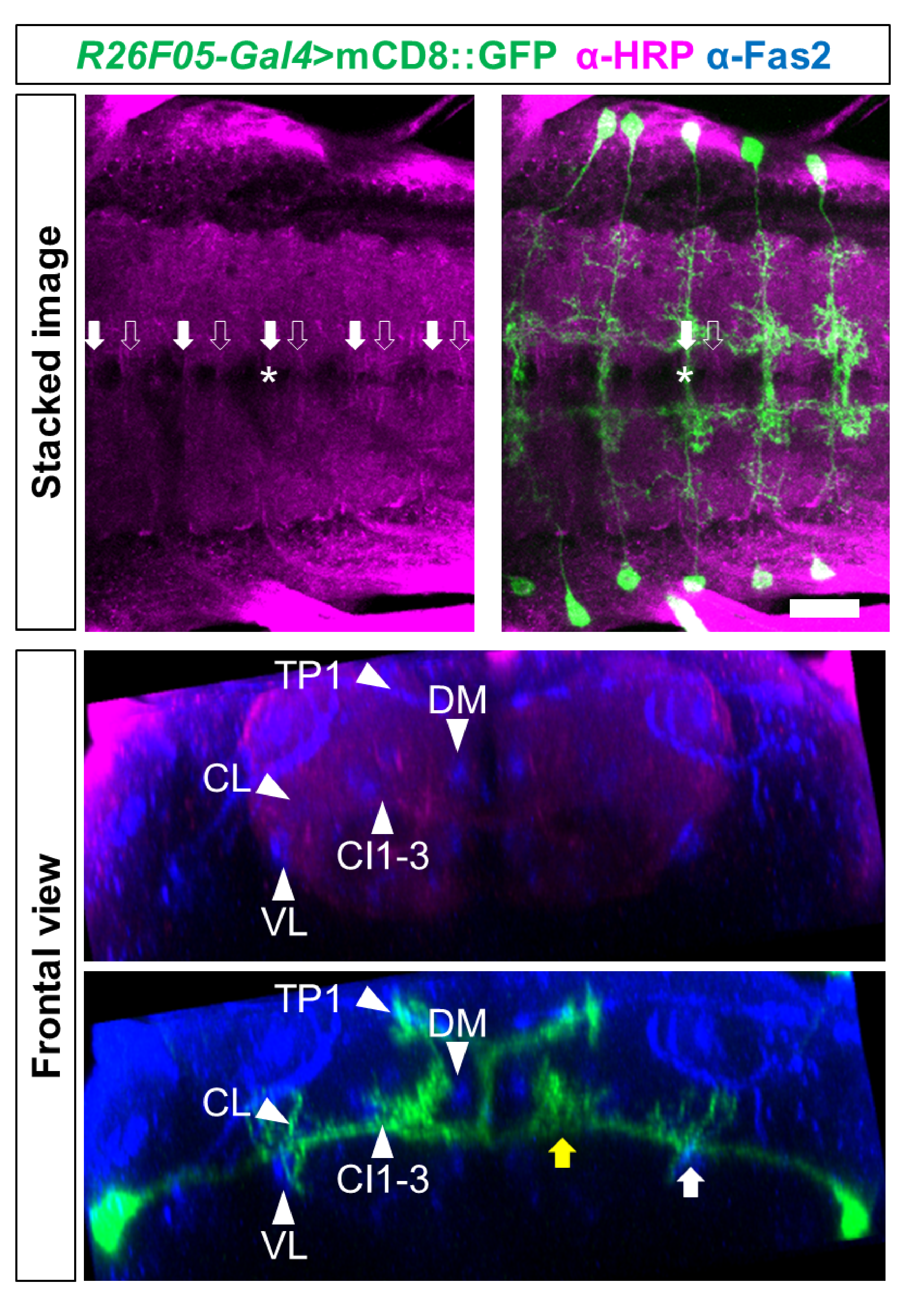

Supplement: S1 Fig — (upper two panels) The morphology of GVLIs (visualized with mCD8::GFP expressed under the control of R26F05-Gal4, green) in relation to the anterior commissure (white arrows) and posterior commissure (open arrows) labeled with anti-HRP antibody (magenta). A2-A6 neuromeres of the ventral nerve cord are shown. Asterisks indicate the position of A4 anterior commissure. Scale bar, 20 μm. (bottom two panels) The morphology of GVLIs (green, labeled as in upper panels) in relation to the Fasciclin2-positive axon tracts (blue, tracts indicated by white arrowheads). The yellow and white arrows correspond to those in Fig 2K, 2M and 2N. (TIF) [file pone.0136660.s001.tif]

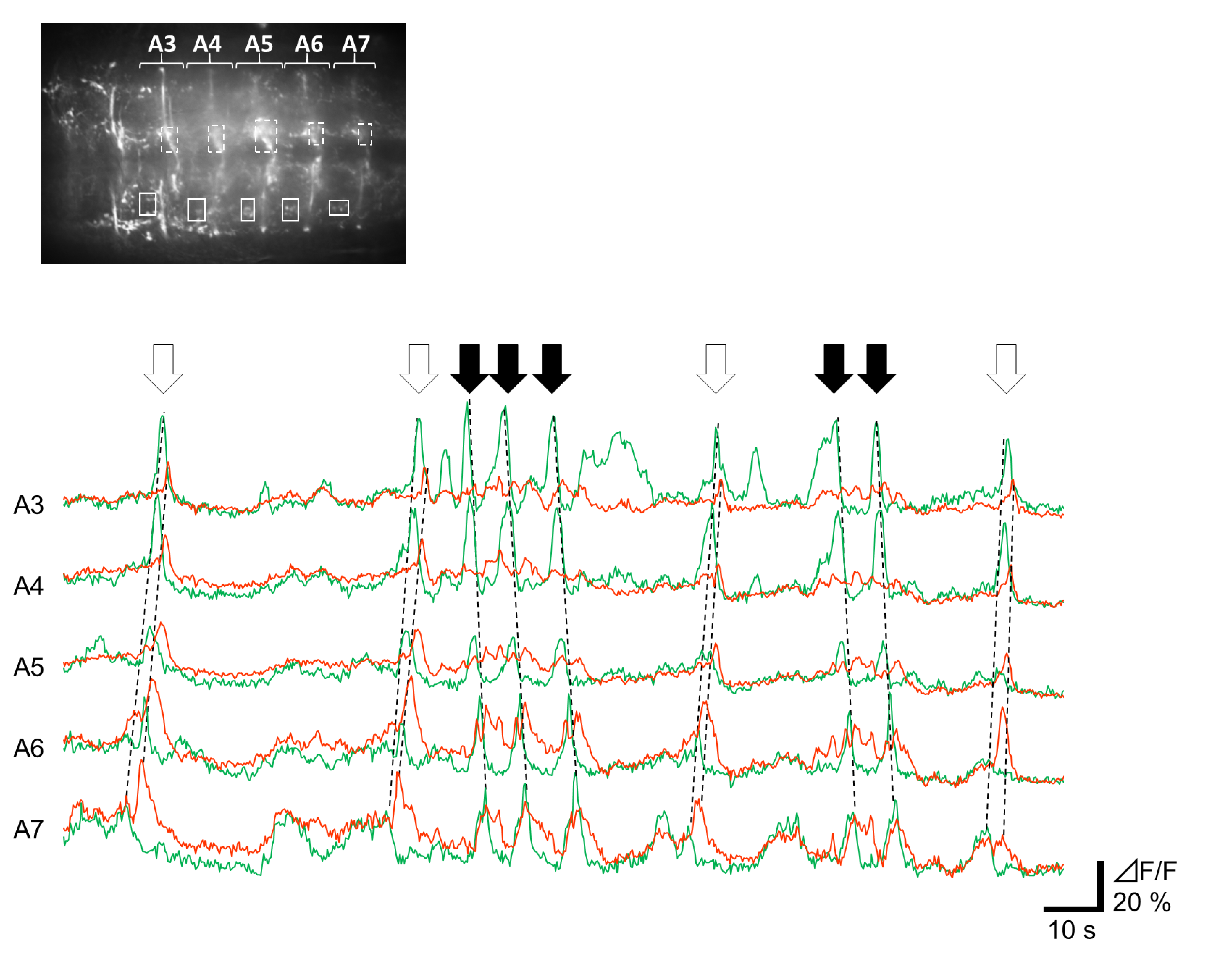

Supplement: S3 Fig — The time courses of GCaMP6f signal intensity change in the neurites of GVLIs (the dotted rectangles and orange lines) and PMSIs (the solid rectangles and green lines) in neuromeres A3-A7 are shown. Activity propagation of GVLIs lagged behind that of PMSIs by ~2 neuromeres during forward waves (white arrows). PMSIs but not GVLIs were activated in a wave-like manner during backward waves (black arrows). ROIs used for the recording are shown in the upper panel. In this representative example, ROIs for GVLIs were set in the right side of the VNC and those for PMSIs were in the left side to minimize the overlap. (TIF) [file pone.0136660.s003.tif]
